# Distinguishing the Concentration- vs. Bioaccumulation-Dependent Immunological and Metabolic Effects of Clofazimine

**DOI:** 10.3390/pharmaceutics15092350

**Published:** 2023-09-20

**Authors:** Andrew R. Willmer, Jennifer Diaz-Espinosa, Austin Zhou, Kathleen A. Stringer, Gus R. Rosania

**Affiliations:** 1 Department of Pharmaceutical Sciences, College of Pharmacy, University of Michigan, Ann Arbor, MI 48109, USA; awillmer@umich.edu (A.R.W.); jendiaz@umich.edu (J.D.-E.); austinyz@umich.edu (A.Z.); 2 Department of Clinical Pharmacy, University of Michigan, Ann Arbor, MI 48109, USA; stringek@umich.edu

**Keywords:** splenectomy, cytokines, macrophages, drug targeting, drug delivery

## Abstract

The antimycobacterial drug clofazimine (CFZ) is used as a single agent at high doses, to suppress the exaggerated inflammation associated with leprosy. Paradoxically, increasing doses of CFZ leads to bioaccumulation of CFZ in the spleen and other organs under physiologically relevant dosing regimens, without accompanying dose-dependent elevation in the concentrations of the circulating drug in the blood. In long-term oral dosing regimens, CFZ induces immunological and metabolic changes resulting in splenomegaly, while the mass of other organs decreases or remains unchanged. As an organ that extensively sequesters CFZ as insoluble drug precipitates, the spleen likely influences drug-induced inflammatory signaling. To probe the role of systemic drug concentrations vs. drug bioaccumulation in the spleen, healthy mice were treated with six different dosing regimens. A subgroup of these mice underwent surgical splenectomies prior to drug treatment to assess the bioaccumulation-dependent changes in immune system signaling and immune-system-mediated drug distribution. Under increasing drug loading, the spleen was observed to grow up to six times in size, sequestering over 10% of the total drug load. Interestingly, when the spleen was removed prior to CFZ administration, drug distribution in the rest of the organism was unaffected. However, there were profound cytokine elevations in the serum of asplenic CFZ-treated mice, indicating that the spleen is primarily involved in suppressing the inflammatory signaling mechanisms that are upregulated during CFZ bioaccumulation. Thus, beyond its role in drug sequestration, the spleen actively modulates the systemic effect of CFZ on the immune system, without impacting its blood concentrations or distribution to the rest of the organism.

## 1. Introduction

Clofazimine (CFZ) is a small-molecule antibiotic with low solubility that is indicated for the treatment of leprosy and is effective against multidrug-resistant tuberculosis [1,2]. At therapeutic doses, CFZ molecules accumulate in the organs of the reticuloendothelial system such as the liver and spleen [3]. Following repeated dosing in mice and humans, CFZ is sequestered by tissue-resident macrophages where it precipitates as crystalline deposits (crystal-like drug inclusions, CLDIs) within acidic endolysosomal organelles [4,5,6]. Lysosomal proton pumps and chloride transporters stabilize the precipitation of CFZ as a hydrochloride salt, making the salt form of the drug in CLDIs even less soluble than its free base form. Soluble CFZ molecules are highly reactive, exhibiting a broad spectrum of antimicrobial activity [7,8,9]. However, crystallized CFZ precipitates have been shown to be well-tolerated with few toxicological effects, and induce an anti-inflammatory state mediated with the interleukin receptor 1 receptor antagonist (IL1-RA) [10,11].

In long-term dosing experiments in mice, CFZ has been shown to influence metabolism, with consequent effects on organ mass and total body weight. As a catabolic agent, CFZ induces weight loss in mice despite modest increases in food consumption relative to vehicle-treated controls [12,13]. Additionally, after 8 weeks of CFZ treatment in mice, the size of the spleen increases dramatically, likely due to increased macrophage recruitment to accommodate CLDI storage [6,14,15]. Because of the increase in macrophage numbers, the organs that see the largest increase in the sequestered drug after repeated dosing are the spleen and liver, organs with high numbers of resident macrophages [2,14]. As the spleen is an important organ in storing CLDIs and modifying immune cell activity, we explored the effects of spleen removal on immune signaling and CFZ tissue distribution in mice during a long-term treatment regimen.

## 2. Materials and Methods

### 2.1. CFZ Dosing, Determination of CFZ Loads, and Splenectomy Experiments in Mice

Animal care was provided by the University of Michigan’s Unit for Laboratory Animal Medicine (ULAM), and the experimental protocol was approved by the Committee on Use and Care of Animals (Protocol PRO00009404). ARRIVE guidelines were followed throughout the duration of this research.

**Figure 1:**
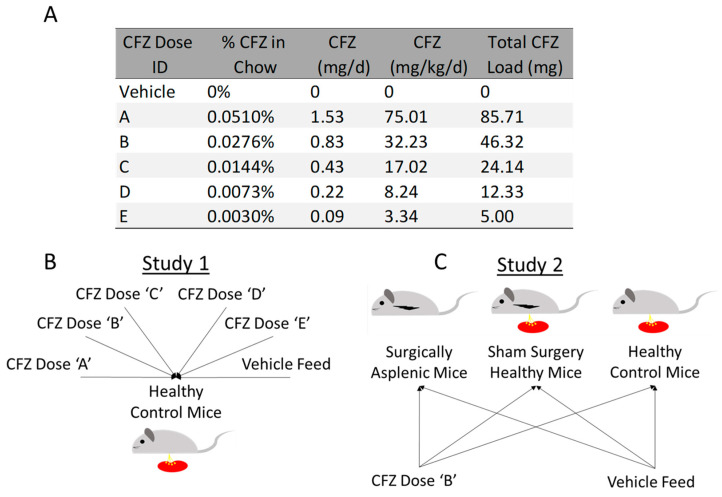
Experimental setup. (**A**) CFZ was administered in one of five dosing regimens. CFZ Dose ‘E’ contained the lowest daily load of CFZ whereas CFZ Dose ‘A’ contained the largest daily dose. Dosing group ‘B’ represents the physiologically relevant dosing scheme used in humans based on allometric scaling conducted in previous work [14,16]. Groups ‘A–E’ and ‘Vehicle’ were treated for 8 weeks. (**B**) For Study 1, mice (*n* = 36; 6 per group) were randomized (1:1) to one of five doses of CFZ or control. (**C**) In Study 2, mice (*n* = 48) were randomized (1:1) to either splenectomy, sham surgery, or no surgical intervention (healthy control mice). Animals within each group were then randomized to either Dose ‘B’ or vehicle (*n* = 8 per group).

Two independent studies were conducted in four-week-old male mice (C57BL/6J, Jackson Laboratory, Bar Harbor, ME). Mice were dosed with either CFZ or vehicle feed for a total of 8 weeks. In Study 1, mice (*n* = 36) were randomized 1:1 into one of five different CFZ treatment groups or a vehicle-fed control group. CFZ was orally administered through milled feed consisting of CFZ dissolved in sesame oil mixed with Powdered Lab Diet 5001, consistent with previously conducted experiments (Figure 1A,B) [14]. Vehicle-treated animals received drug-free sesame oil mixed with milled feed. A preliminary pilot study was conducted to assess changes in the spleen and total body weight of mice treated with CFZ for 65 weeks; these mice were categorized as group ‘X’ and only used for comparison of spleen and total body weight. Previous studies determined that mice consume approximately 3 g of feed per day [14]. The daily drug load was then calculated based on the concentration of CFZ mixed into the feed multiplied by the total daily food consumption (Figure 1A). The total drug load was estimated based on a total of 8 weeks of treatment multiplied by the daily drug load. 

In Study 2, mice were randomized into one of six groups, drug-treated splenectomy, drug-treated sham, drug-treated control, vehicle-treated splenectomy, vehicle-treated sham, or vehicle-treated control (Figure 1C), with eight mice per group. Mice that underwent surgery were anesthetized with isoflurane and surgery was performed under sterile conditions. Following a small abdominal incision, the spleen was retrieved from the upper left abdominal quadrant, and either removed (splenectomy group) or returned into the abdominal cavity (sham-surgery group). The abdominal cavity was then closed with interrupted sutures, and the skin was closed with surgical glue. Mice received 5 mg/kg of subcutaneous carprofen daily for 48 h post-surgery, and a one-time dose of subcutaneous lidocaine, 2 mg/kg, prior to surgery; they were monitored daily. After 10 days of postoperative recovery, mice were treated with either CFZ or a drug-free vehicle for 8 weeks as described above. The drug diet was administered for 8 weeks, after which the mice were euthanized for tissue collection.

### 2.2. Sample Preparation and Measurements of CFZ Concentrations

The whole blood, lung, liver, kidney, heart, and spleen were harvested from mice at the end of the 8-week dosing period. Whole blood was collected from mice at the time of euthanasia with a cardiac puncture. The blood was allowed to clot for 30 min, then serum was generated with centrifugation. The lung, liver, kidney, and spleen were harvested, rinsed with PBS, and blotted dry prior to freezing. Organs and serum were then flash frozen in liquid nitrogen and stored (−80 °C) until the time of the analysis. At the time of a cytokine assay, serum was thawed on ice and 100 µL was used for the measurement of cytokine concentrations. The remaining volume of the serum was refrozen using liquid nitrogen and stored (−80 °C) for the measurement of CFZ concentration. 

Serum and organ CFZ concentrations were analyzed with liquid-chromatography–mass-spectrometry (LC-MS) at the University of Michigan’s Pharmacokinetic Core, and skin concentrations were analyzed with absorbance spectroscopy as previously described [17]. Tissue samples were weighed and homogenized with either 10 times (spleen and lung) or 15 times (liver) of the volume of an 80% N-Dimethylformamide (DMF)-PBS solution, and vortex mixed. No dilution of lung samples was conducted, but liver and spleen samples were further diluted 20 times with 1X PBS and vortex mixed. Using our established CFZ liquid chromatography (LC)–mass spectrometry (MS) assay [14], a calibration curve was prepared with a concentration range from 1 to 50 µg/mL of CFZ in respective blank tissues. Analytical curves were constructed by plotting the peak area ratio of CFZ to the internal standard versus the concentration using linear regression with weighting (1/X or 1/X^2^). The concentration range was evaluated from 1 to 50 µg/mL for quantification with LC-MS. A blank sample (matrix sample processed without internal standard) was used to exclude contamination or interference. 

### 2.3. Measurement of Cytokine Concentrations in Serum

Serum cytokines were measured using a commercially available assay (Mouse cytokine array panel A, R&D ARY006 kits, Minneapolis, MN, USA) [18]. Serum (100 µL) from each mouse was diluted, placed on array membranes, and incubated with detection antibodies for 24 h per the manufacturer’s instructions. After incubation, membranes were washed and treated with chemiluminescent reagents for imaging. The pixel density of each cytokine signal was evaluated with an iBright FL1500 imaging system (Invitrogen, Thermo Fisher Scientific, Waltham, MA, USA). Duplicate analyte signals were averaged before subtracting the background signal, after which they were normalized to the positive control on each individual membrane. The resulting value is referred to as the relative density and is used to compare relative cytokine concentrations between experimental groups [14].

### 2.4. Statistical Analysis

Organ and serum CFZ concentrations, as well as organ weights, were compared using an ANOVA with Tukey’s post hoc analysis to account for multiple comparisons when applicable. Differences in cytokine concentrations among all groups in Study 2 were evaluated using an ANOVA with Cohen’s D test followed by Tukey’s post hoc analysis. Significance was determined with *p* < 0.05. The percent difference and effect size were used to determine the magnitude of difference between cytokine concentrations of different experimental groups. To determine the relative effect of CFZ in asplenic mice, cytokine concentrations in the CFZ-treated asplenic mice were normalized to the concentrations of vehicle-treated asplenic mice, and the CFZ-treated sham-surgery mice were normalized to the concentrations of vehicle-treated sham-surgery mice. The resulting normalized groups were then compared as previously described.

## 3. Results

### 3.1. CFZ Induces Dose-Dependent Catabolic and Splenomegaly Phenotype

**Figure 2:**
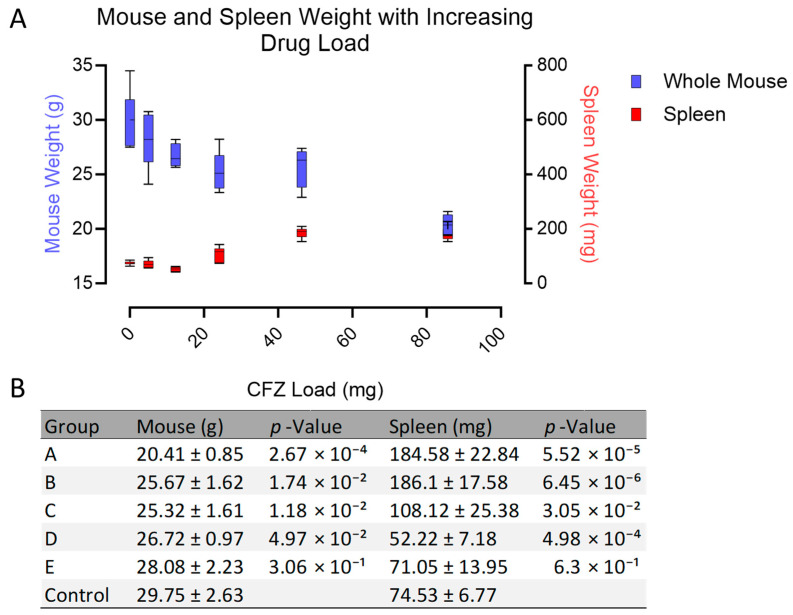
CFZ induced changes in organ mass with increasing drug concentration. (**A**) Spleen weight (red) and total body weight (blue) are plotted across increasing drug load. The colored boxes represent the 25th and 75th percentile with the horizontal line splitting the box representing the median. The whiskers correspond to 10th and 90th percentile (*n* = 6 per group). (**B**) Mean (±S.D.) total mouse body weight (g) and spleen weight (mg) are listed across each dosing group (A–E and control). *p*-Value was determined with an unpaired Student’s *t*-test using the control group as the comparator. For comparisons among all groups and organ systems, ANOVA with Tukey post hoc analysis was completed and is shown in the Supplementary Materials (Tables S1 and S2).

The catabolic effect resulting from CFZ treatment was evident in all measured organ systems except for the spleen in which a profound increase in mass is observed (Figure 2 and Tables S1 and S2). After 8 weeks of dosing, there was no increase in spleen mass nor decrease in the mass of other organs observed in the group of animals given the lowest CFZ doses (groups D and E) compared to the vehicle-treated control group. This suggests that the dose of CFZ in groups D and E was too small to cause splenomegaly or catabolism. However, at a total load of 24 mg (group C), the spleen mass increased as the precipitated drug load accumulated within the spleen macrophages, up until 42 mg (group B), at which point the increase in spleen mass plateaued up to a CFZ load of 84 mg (group A). The total body weight continued to decrease with an increased total drug load, indicating that the loss of body mass was not necessarily linked to the enlargement of the spleen. Most significantly, the group with the highest drug load (group A) showed a substantial decrease compared to group B, the second highest drug load (20.41 ± 0.85 vs. 25.67 ± 1.62, *p* = 0.0007). When compared to group B, the spleen size of group ‘X’ (treated for 65 weeks) increased over three-fold when given the same dose over an extended period of time (186.1 g vs. 596.2 g, *p* = 0.007). Collectively, these findings indicate that spleen size and total body weight are not exclusively determined with the load of the drug, but are the result of complex, time, and dose-dependent physiological changes in metabolism and immune function. 

### 3.2. Pharmacokinetic Analysis of CFZ with Increasing Drug Load and Asplenia

**Figure 3:**
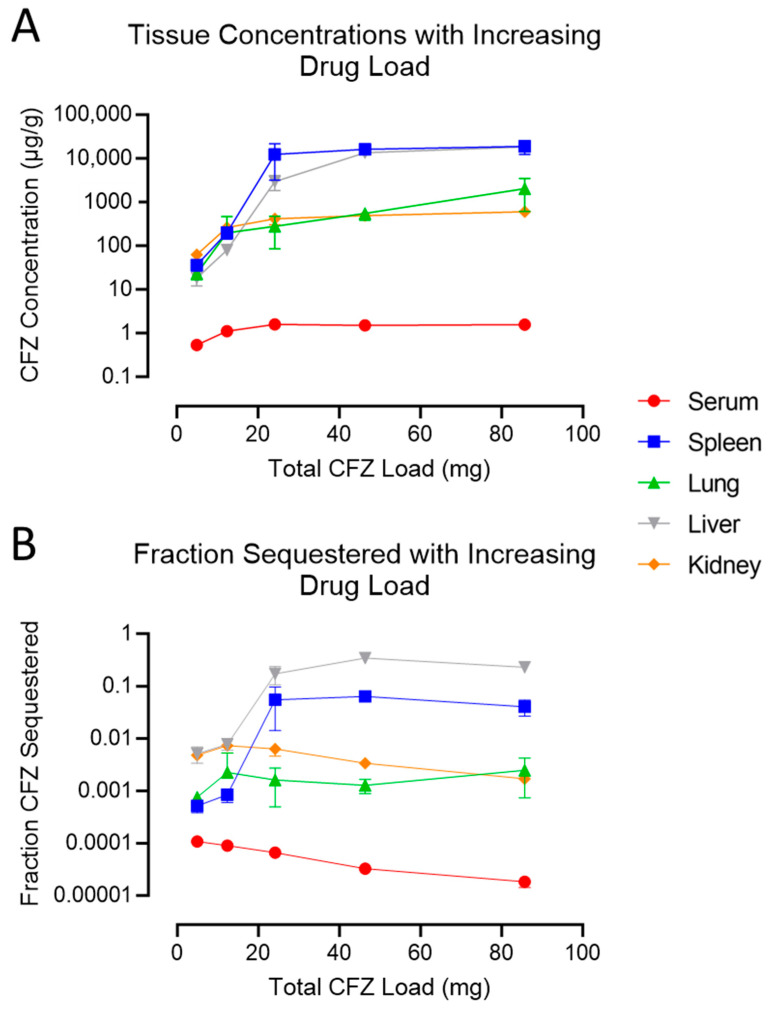
CFZ distribution with increasing drug load. (**A**) Concentration of CFZ in each organ was plotted with increasing drug load. (**B**) Fraction of total administered drug sequestered in each organ was plotted with increasing drug load. Fraction sequestered was determined by dividing the mass measured in each organ by the total amount of drug administered over the 8-week dosing duration. Circulating serum was estimated to be 1000 µL in volume (*n* = 6 per group). Numerical averages, standard deviations, and *p*-values (calculated using ANOVA with Tukey post hoc analysis) are shown in the Supplementary Materials (Tables S3–S6).

As the total administered CFZ load increased, the measured drug concentration in every organ system increased in parallel (Figure 3A and Tables S3 and S4), yet after a total load of 24 mg, the serum concentrations of CFZ were dose-independent, plateauing at 1 µg/mL, consistent with literature values [2] and as predicted with pharmacokinetics models incorporating a soluble-to-insoluble phase transition driven by drug precipitation in macrophage lysosomes [19,20]. Notably, only the serum and kidney concentrations increased between group E and group D, indicating that the increase in drug load at low doses only impacted the serum and kidney concentrations, and not those of the spleen, lung, or liver.

All solid organ systems continued to sequester increasing concentrations of the drug as the total load of the drug administered to the mice reached 42 mg. When the total load of the drug administered was increased from 42 to 84 mg (group B to group A), CFZ bioaccumulation reached a maximum in every organ except for the lungs and liver, which continued to accumulate the drug. However, when accounting for organ mass, only the liver showed an increase in sequestered CFZ between groups B and A (Figure S1).

**Figure 4:**
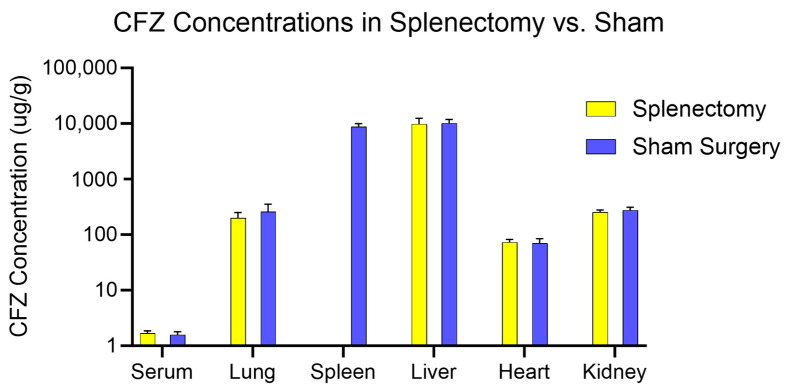
CFZ concentrations in organs from mice that underwent splenectomy (yellow) compared to mice that received sham surgery (blue). There were no significant differences in CFZ serum or organ concentrations between splenectomy and sham-surgery mice (*p* < 0.05). N = 8 per group.

To explore the role of the spleen in the immunomodulatory effects of CFZ and its systemic biodistribution behavior, we employed the physiologically relevant dosing scheme ‘B’ for experiments in Study 2. Surprisingly, there were no differences in CFZ organ concentrations from asplenic mice compared to the sham-surgery (control) CFZ-treated animals (Figure 4). Additionally, neither spleen removal nor sham surgical intervention impacted the concentration of CFZ in the skin (Figure S2).

In splenectomized mice, there were no changes in drug concentrations in other major organs of the reticuloendothelial system, when compared to the CFZ distribution in the sham-surgery (control) group of mice (Figure S3). Furthermore, there were no differences in organ weights between these two groups, indicating that the spleen removal did not affect the CFZ-induced catabolic state that paralleled the increase in the daily dose of CFZ (Figure S4). This implies that the catabolism observed upon CFZ administration occurs independently from the changes in spleen mass, and independent of the physiological functions of the spleen. Additionally, there were no differences in the concentration or total mass of bioaccumulated CFZ between the splenectomized mice and the CFZ-treated controls (Figures S5 and S6).

### 3.3. CFZ-Induced Changes in Cytokine Levels in Asplenic Mice

**Figure 5:**
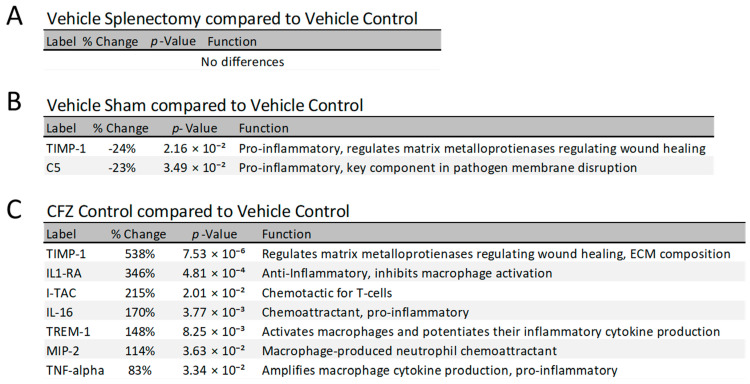
Cytokine analysis compared to control. After adjusting for multiple comparisons, significant changes in cytokine concentrations were recorded for (**A**) vehicle splenectomy compared to vehicle control, (**B**) vehicle sham compared to vehicle control, and (**C**) CFZ control compared to vehicle control. Cytokine functions [21,22,23,24,25,26,27,28] are listed alongside the percent difference and *p*-value.

Previous work has shown that the increasing CFZ sequestration in CLDIs results from proliferation of macrophage populations, leading to an enlarged total volume of distribution to sequester the drug [15]. In addition, proteomics experiments have identified the cytokine profile of different organs, yielding valuable insights into the mechanism of CFZ-induced macrophage proliferation and the anti-inflammatory effect of CFZ [11,14]. To determine whether CFZ bioaccumulation influences systemic immune signaling molecules, serum cytokine profiles from both vehicle-treated and CFZ-treated splenectomized and sham-surgery mice were compared to their respective controls without surgical intervention. There were few significant differences in cytokine concentrations when assessing the impact of surgical intervention alone (Figure 5A,B), indicating both splenectomy and sham surgery had little to no impact on the cytokine profiles after 8 weeks post-operation. 

**Figure 6:**
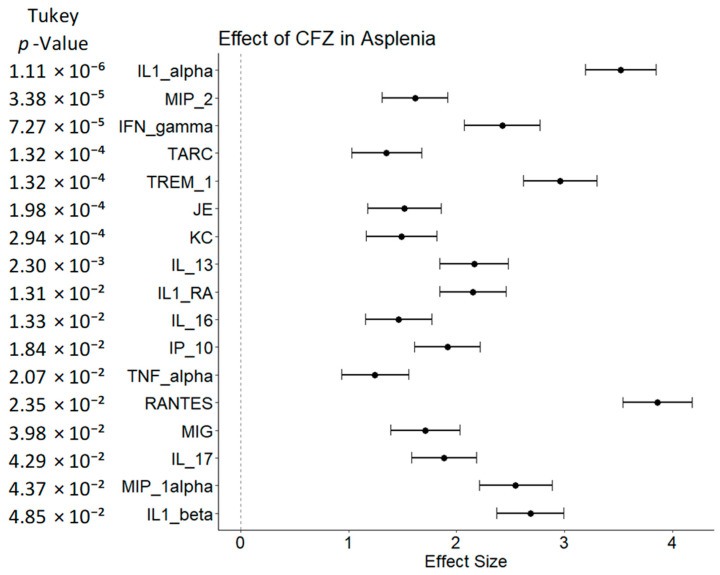
Normalized relative density of serum cytokine levels between the CFZ-treated splenectomy mice and CFZ-treated sham-surgery mice. Effect size was calculated using Cohen’s D. Multiple comparisons were accounted for with Tukey’s post hoc analysis (shown *p*-values). Effect size > 0 indicates more activity in the splenectomy group, and effect size < 0 indicates more activity in the sham group (*n* = 8 in each comparator group). Only the cytokines with significant differences between the two groups were plotted. IL = Interleukin, MIP = Macrophage inflammatory protein, IFN = Interferon, TARC = Thymus and activation regulated chemokine, TREM = Triggering receptor expressed on myeloid cells, JE = monocyte chemoattractant protein, KC = Keratinocyte chemoattractant, IL1-RA = Interleukin 1 receptor antagonist, TNA = Tumor necrosis factor, RANTES = Regulated upon activation; normal T-cell expressed and secreted, MIG = Monokine induced by gamma interferon.

There were no differences in serum cytokine levels between the vehicle-treated splenectomy (Figure 5A) and vehicle control, and there were only modest differences in the vehicle-treated sham surgery compared to vehicle control (no surgery; Figure 5B). However, CFZ treatment alone showed dramatic increases to tissue inhibitor matrix metalloproteinase 1 (TIMP-1) and IL1-RA (538% and 346% increases, respectively), alongside a modest increase in TNF-alpha (83%) and several other pro- and anti-inflammatory cytokines (Figure 5C). When normalized to their respective controls, CFZ had a profound impact on the cytokine levels in asplenic mice compared to CFZ-treated sham-surgery mice (Figure 6), far exceeding the expected results with either CFZ or asplenia alone.

Of the 40 measured cytokines, 17 demonstrated increased expression in the CFZ splenectomy group normalized to the CFZ sham-surgery control. These analytes consisted of both pro- and anti-inflammatory immune signaling molecules within the CFZ-treated, splenectomized animals. Of particular interest, five of the seven upregulated cytokines from the CFZ treatment group were amplified in mice that had their spleen removed prior to the 8-week CFZ dosing regimen (MIP-2, TREM-1, IL1-RA, IL-16, and TNF-alpha), indicating that the spleen serves to decrease levels of immune signaling molecules that are upregulated with CFZ treatment. Average density values and corresponding standard deviations for all cytokines across each group in Study 2 are reported in Table S7.

## 4. Discussion

While limited research has been performed on the role of the spleen in drug pharmacokinetics and pharmacodynamics, tens of thousands of splenectomies are conducted within the US annually [29]. Hence, we decided to explore the impact of splenectomy on drug disposition and associated changes in systemic cytokine levels, starting with a drug that is known to extensively bioaccumulate in the spleen [11,30,31,32,33,34,35]. By performing splenectomy in mice before treating them with CFZ, at clinically relevant doses [14,16], we sought to elucidate differences in CFZ distribution that could result from the removal of this organ. While it is already known that asplenic individuals are more susceptible to infection [36], the role of the spleen as a mediator of drug-induced changes in immune signaling, pharmacokinetics, and metabolic drug side effects has not been previously studied. 

Primarily, the increase in serum immune signaling molecules that followed CFZ treatment in asplenic mice is consistent with a splenic downregulation of circulating cytokines that are upregulated with CFZ bioaccumulation in resident tissue macrophages. Previous studies have shown immune dysregulation caused by asplenia and hyposplenia can lead to overwhelming post-splenectomy infection, or other immune-mediated pathologies such as celiac disease [37]. In the case of splenectomized mice, there are several other pro- and anti-inflammatory cytokines that are upregulated only upon CFZ treatment, which lends further support to the notion that the spleen is specifically inhibiting the immunological response to the drug. The difference in systemic cytokine activity between splenectomy and sham-surgery groups highlights the importance of the spleen in maintaining immune system homeostasis during CFZ treatment, indicating that the observed changes are not simply the result of the stress imposed with surgical intervention. Post-splenectomy, the introduction of CFZ resulted in cytokine signaling consistent with M2 macrophage differentiation [26,27,38,39]. Prior examination of macrophage populations after CFZ dosing has shown increases in monocyte proliferation, liver granulomas, and profound splenomegaly, indicative of increased immune activity [14,40]. The relative increase in serum cytokine levels of the CFZ-treated splenectomy group compared to CFZ alone shows that asplenia plays a role in furthering this response.

In terms of the specific functions of the immune signaling molecules affected by splenectomy in CFZ-treated mice, TREM-1, IL-16, and TNF-alpha have a prominent role in macrophage proliferation [26,27], and IL1-RA, while classically thought of as an anti-inflammatory cytokine, aids in the differentiation of M2 macrophages specializing in tissue healing and injury repair. These macrophages upregulate more anti-inflammatory cytokines such as TREM-1, and IL-13, which were observed in increased concentrations in the CFZ-treated asplenic mice compared to the CFZ-treated sham-surgery controls [26,27,38]. Collectively, these changes are associated with increased macrophage proliferation in the CFZ-treated asplenic mice with a propensity to specialize as anti-inflammatory M2 macrophages. Of particular interest, IL-1 alpha, an immune protein important in macrophage recruitment and a marker of macrophage stress [39], had the most significant increase in the asplenic, CFZ-treated mice when compared to the sham-surgery control. This finding implies that the spleen modulates the magnitude of the CFZ inflammatory response, and in the absence of splenic modulation, a more profound immune response to CFZ is observed. Prior research has established massive drug accumulation in macrophages, leading to increases in the macrophage number and the formation of liver granulomas [5,11,14,40]. However, studies on macrophage populations and complete blood counts are an important next step in identifying a causal link between splenectomies and immune cell differentiation in the presence of CFZ.

CFZ pharmacokinetics have been analyzed in detail in previous work [19,20], showing that as CFZ is sequestered by resident macrophages, there is a large increase in total macrophage populations, accompanied by changes in circulating and organ-specific cytokine levels [15]. Cytokines are a group of inflammatory mediators that control the immune responses to external stimuli [41]. Upon injury or infection, cytokines initiate defense and repair mechanisms to protect and restore tissue health [42]. The systemic levels of cytokines can indicate the prevalence and function of immune cells, such as macrophages, that produce these cytokines [43,44]. CFZ exerts anti-inflammatory effects when sequestered by macrophages such as increased expression of IL-1RA, which may serve to dampen the pro-inflammatory effects of soluble CFZ by inhibiting the effect of proinflammatory cytokines such as tumor necrosis factor alpha (TNF-alpha) and other pro-inflammatory molecules [14,23,24].

## 5. Conclusions

To summarize, the accumulation of CFZ in the spleen of CFZ-treated animals occurs independently from the concentration of the drug in the serum with increasing daily doses of the administered drug. With increasing total administered loads of CFZ, the spleen increased in mass but CFZ concentrations in the blood remained comparable to the blood concentrations of mice that did not show significant spleen enlargement. As the total drug load increased, there was also an increase in the catabolic effect of CFZ, which was independent of the concentrations of the drug in the blood stream. Thus, the catabolic effect of CFZ, as well as the increase in spleen mass that results from CFZ treatment, both remain dependent on the total drug load and independent of the concentration of the drug in systemic circulation. 

In terms of the role of the spleen in CFZ-mediated changes in immune signaling, CFZ-treated asplenic mice survived the drug treatment, had no discernable adverse events, and did not exhibit differences in the sequestered mass of CFZ in every measured organ as compared to the CFZ-treated sham-surgery control mice. Thus, there were no overt signs of toxicity resulting from removal of this major drug sequestering organ. In healthy mice, the spleen acted as a drug depot organ for CFZ. However, in splenectomized mice, there were no changes in drug distribution to other organs of the reticuloendothelial system that would have compensated for the absence of the spleen. The removal of the spleen increased circulating levels of cytokines after drug administration compared to the CFZ-treated sham-surgery control, which implicates the spleen as a modulator of the immune response from CFZ treatment. Lastly, it is worth noting that CFZ shares with other lysosomotropic drugs a macrophage-dependent accumulation mechanism. While these experiments were performed in mice, the results highlight how asplenic patients treated with CFZ could yield additional insights into the mechanism of action and side effects of this and other bioaccumulating drugs [45].

## Data Availability

Not applicable.

## Supplementary Materials

The following supporting information can be downloaded at: https://www.mdpi.com/article/10.3390/pharmaceutics15092350/s1, Table S1: Averages and standard deviations for the mice and organ weights for Study 1; Table S2: *p*-Values for Weight Comparisons; Table S3: Averages and standard deviations for the CFZ concentrations in each organ for Study 1; Table S4: *p*-Values for CFZ Concentration Comparisons; Table S5: Averages and standard deviations for the fraction of drug sequestered in each organ for Study 1; Table S6: *p*-Values for Fraction of Drug Sequestered; Table S7: Cytokine Density Values; Figure S1: Total CFZ mass by organ; Figure S2: Quantitative analysis of CFZ concentration in the skin after 8 weeks of CFZ treatment; Figure S3: Drug sequestration in asplenic mice; Figure S4: Weights of the primary drug sequestering organs in Study 2; Figure S5: CFZ concentration comparisons in the primary drug sequestering organs; Figure S6: Total CFZ mass comparisons among the primary drug sequestering organs.
