# Supplementary material for: Distinguishing the Concentration- vs. Bioaccumulation-Dependent Immunological and Metabolic Effects of Clofazimine
Source: Pharmaceutics. 2023 Sep 20;15(9):2350. doi: 10.3390/pharmaceutics15092350 (PMC10537750; doi:10.3390/pharmaceutics15092350)
Supplement: Supplementary file 1 [file pharmaceutics-15-02350-s001.zip › pharmaceutics-2593699-supplementary.pdf]

Supplementary Materials

S.1 Study 1 Mouse Organ Weights and Drug Concentrations

Table S1: Averages and standard deviations for the mice and organ weights for study 1.

Group	Mouse (g)	Liver (mg)	Spleen (mg)	Lung (mg)	Kidney (mg)
A	20.41 ± 0.85	1065.88 ± 129.14	184.58 ± 22.84	104.02 ± 19.62	380.47 ± 38.03
B	25.67 ± 1.62	1166.08 ± 88.77	186.1 ± 17.58	106.68 ± 6.7	400.42 ± 38.86
C	25.32 ± 1.61	1408.92 ± 167.41	108.12 ± 25.38	139.68 ± 23.47	244.18 ± 27.41
D	26.72 ± 0.97	1206.4 ± 130.7	52.22 ± 7.18	140.33 ± 35.48	322.75 ± 23.4
E	28.08 ± 2.23	1388.23 ± 130.69	71.05 ± 13.95	162.23 ± 25.36	366.58 ± 29.26
Control	29.75 ± 2.63	1369.18 ± 172.08	74.53 ± 6.77	160.73 ± 20.13	343.58 ± 22.91

Table S2: P-Values for Weight Comparisons. P-values and the level of statistical significance were calculated after ANOVA with Tukey's post hoc analysis for each measured organ system between each dosing regimen (A-E), with n=6/group.

P-Values for Weight Comparisons

Comparison	Total		Spleen		Lung		Liver		Kidney	
A vs. B	0.0007	***	>0.9999	ns	>0.9999	ns	0.8617	ns	0.0041	**
A vs. C	0.0016	**	<0.0001	****	0.1852	ns	0.0062	**	<0.0001	****
A vs. D	<0.0001	****	<0.0001	****	0.1707	ns	0.6077	ns	0.0002	***
A vs. E	<0.0001	****	<0.0001	****	0.0056	**	0.0113	*	<0.0001	****
A vs. Control	<0.0001	****	<0.0001	****	0.0073	**	0.0193	*	<0.0001	****
B vs. C	0.9996	ns	<0.0001	****	0.2546	ns	0.0931	ns	0.241	ns
B vs. D	0.9322	ns	<0.0001	****	0.2362	ns	0.9972	ns	0.8875	ns
B vs. E	0.2854	ns	<0.0001	****	0.0089	**	0.1496	ns	0.0577	ns
B vs. Control	0.0115	*	<0.0001	****	0.0115	*	0.2231	ns	0.0046	**
C vs. D	0.808	ns	0.0002	***	>0.9999	ns	0.2257	ns	0.8397	ns
C vs. E	0.1653	ns	0.0207	*	0.6526	ns	0.9999	ns	0.9784	ns
C vs. Control	0.0052	**	0.0443	*	0.7138	ns	0.9974	ns	0.5141	ns
D vs. E	0.8243	ns	0.5204	ns	0.6794	ns	0.3321	ns	0.42	ns
D vs. Control	0.103	ns	0.3363	ns	0.7394	ns	0.4519	ns	0.0639	ns
E vs. Control	0.673	ns	0.9995	ns	>0.9999	ns	>0.9999	ns	0.9043	ns

*ns = not significant, * p<0.05, **p<0.01, ***p<0.001, ****p<0.0001*

Table S3: Averages and standard deviations for the CFZ concentrations in each organ for study 1.

Group	Serum (ug/mL)	Spleen (ug/g)	Lung (ug/g)	Liver (ug/g)	Kidney (ug/g)
A	1.58 ± 0.35	18952.5 ± 6527.47	2043.33 ± 1435.62	18387.5 ± 942.17	601.67 ± 79.43
B	1.53 ± 0.16	16055 ± 1436.56	555 ± 167.43	13550 ± 1354.81	488.75 ± 58.88
C	1.6 ± 0.25	12422.83 ± 9239.16	280.08 ± 194.5	2941.67 ± 1097.12	417.5 ± 114.21
D	1.11 ± 0.16	200.83 ± 58.45	197.97 ± 268.89	79.98 ± 18.99	263.92 ± 40.79
E	0.54 ± 0.06	36.07 ± 9.37	23.09 ± 6.25	18.34 ± 6.29	62.67 ± 10.55
Control	< 0.01	1.48 ± 0.16	0.15 ± 0.25	0.05 ± 0.09	0.17 ± 0.08

Table S4: P-Values for CFZ Concentration Comparisons. P-values and the level of statistical significance were calculated after ANOVA with Tukey's post hoc analysis for each measured organ system between each dosing regimen (A-E), with n=6/group.

P-Values for Concentration Comparisons

Comparison	Serum	Spleen	Lung	Liver	Kidney
E vs. D	0.0012 **	>0.9999 ns	0.9905 ns	>0.9999 ns	0.0004 ***
E vs. C	<0.0001 ****	0.0041 **	0.9608 ns	<0.0001 ****	<0.0001 ****
E vs. B	<0.0001 ****	0.0009 ***	0.6399 ns	<0.0001 ****	<0.0001 ****
E vs. A	<0.0001 ****	<0.0001 ****	0.0002 ***	<0.0001 ****	<0.0001 ****
D vs. C	0.0072 **	0.0047 **	0.9995 ns	<0.0001 ****	0.0069 **
D vs. B	0.027 *	0.001 ***	0.8816 ns	<0.0001 ****	<0.0001 ****
D vs. A	0.0096 **	<0.0001 ****	0.0005 ***	<0.0001 ****	<0.0001 ****
C vs. B	0.9797 ns	0.8238 ns	0.9504 ns	<0.0001 ****	0.4181 ns
C vs. A	>0.9999 ns	0.2402 ns	0.0009 ***	<0.0001 ****	0.001 **
B vs. A	0.9916 ns	0.9129 ns	0.0055 **	<0.0001 ****	0.0686 ns

*ns = not significant, * p<0.05, **p<0.01, ***p<0.001, ****p<0.0001*

Between the lowest dose (group E) and the highest dose (group A), the fraction of drug sequestered increased in the spleen and liver, decreased in the serum and kidney, and remained constant in the lung (Figure 3B, Tables S5-S6). These results point to a pattern of distribution which changes in a tissue dependent fashion as drug load increases.

Table S5: Averages and standard deviations for the fraction of drug sequestered in each organ for study 1.

Group	Serum (ug/mL)	Spleen (ug/g)	Lung (ug/g)	Liver (ug/g)	Kidney (ug/g)
A	0.000018 ± 0.000004	0.04081 ± 0.01406	0.00248 ± 0.001742	0.2287 ± 0.01172	0.001714 ± 0.000226
B	0.000033 ± 0.000004	0.06451 ± 0.005772	0.001278 ± 0.000385	0.3411 ± 0.03411	0.003406 ± 0.00041
C	0.000066 ± 0.000011	0.05563 ± 0.04138	0.001621 ± 0.001125	0.1717 ± 0.06403	0.00634 ± 0.001734
D	0.00009 ± 0.000013	0.000851 ± 0.000248	0.002253 ± 0.00306	0.007825 ± 0.001857	0.007354 ± 0.001136
E	0.000109 ± 0.000013	0.000519 ± 0.000135	0.000851 ± 0.000248	0.05563 ± 0.04138	0.06451 ± 0.005772

Table S6: P-Values for Fraction of Drug Sequestered. P-values and the level of statistical significance were calculated after ANOVA with Tukey's post hoc analysis for each measured organ system between each dosing regimen (A-E), with n=6/group.

P-Values for Fraction Sequestered Comparisons

Comparison	Serum		Spleen		Lung		Liver		Kidney	
E vs. D	0.021	*	>0.9999	ns	0.5383	ns	>0.9999	ns	0.002	**
E vs. C	<0.0001	****	0.0009	***	0.8953	ns	<0.0001	****	0.1063	ns
E vs. B	<0.0001	****	0.0006	***	0.9821	ns	<0.0001	****	0.143	ns
E vs. A	<0.0001	****	0.0185	*	0.4008	ns	<0.0001	****	0.0002	***
D vs. C	0.002	**	0.001	**	0.9635	ns	<0.0001	****	0.4371	ns
D vs. B	<0.0001	****	0.0006	***	0.8465	ns	<0.0001	****	<0.0001	****
D vs. A	<0.0001	****	0.0197	*	0.9993	ns	<0.0001	****	<0.0001	****
C vs. B	<0.0001	****	0.9607	ns	0.9963	ns	<0.0001	****	0.0003	***
C vs. A	<0.0001	****	0.7211	ns	0.8965	ns	0.0435	*	<0.0001	****
B vs. A	0.1052	ns	0.4018	ns	0.7229	ns	<0.0001	****	0.0566	ns

*ns = not significant, * p<0.05, **p<0.01, ***p<0.001, ****p<0.0001*

S.2 Organ Dependent CFZ Mass Calculations in Study 1

Mass balance was conducted to ensure no gross contamination or analytical error occurred during the experiment. CFZ mass was calculated in each group by multiplying the concentration of CFZ in the organ by the volume of the respective organ (assuming an organ density of 1 g/mL). Compared to dosing group B, the total drug mass in the liver was significantly lower in groups C, D, and E, and significantly higher in group A. Other than the liver, no other organ system sequestered a significantly different drug mass between groups A and B, (Figure S1). Additionally, both the liver and spleen had large increases in drug mass from group C to B indicating that this jump in drug load corresponded to a selective increase in drug distribution to these organs.

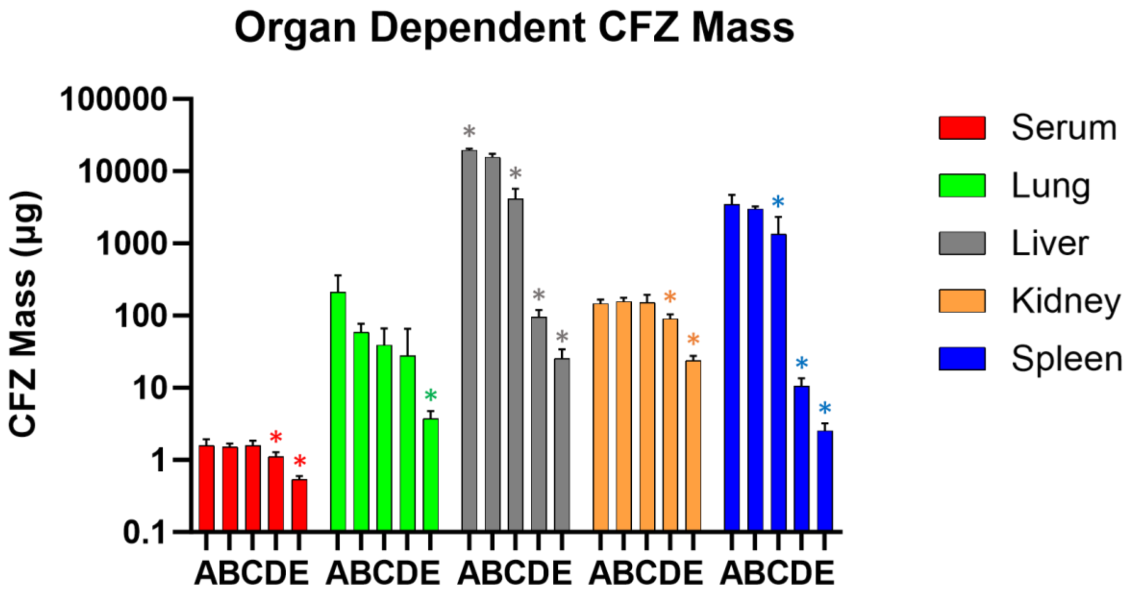

Figure S1: Total CFZ Mass by Organ. CFZ mass in each of the five dosing schemes are compared to the physiologically relevant dosing group B for the serum, lung, liver, kidney, and spleen.

** Significance was determined with p<0.05 by comparison to dosing group B with an unpaired Student's t-test, N=6 in groups A-E.*

S.3 Skin Absorbance of Clofazimine (CFZ)

The high lipophilicity of clofazimine leads to profound partitioning into the skin, causing pigmentation [1]. To assess CFZ distribution to the skin, relative changes in CFZ induced pigmentation were evaluated in mouse ears. Previous studies have demonstrated that CFZ concentration in the skin can be assessed through absorption wavelengths which correspond to different phases of CFZ deposition. The free base form of CFZ was shown to maximally absorb visible light at 450 nm, while the hydrochloride salt of CFZ has a shifted visible absorbance peak at 495 nm. The extent of clofazimine accumulation in the skin was evaluated in mice undergoing surgical asplenia. Four available single-band bandpass optical filters were placed onto an iPhone 13 camera lens for image acquisition. These optical filters screened light at 450 nm, 485 nm, 528

nm, and 620 nm. The flash, high dynamic range (HDR), and night mode options on the camera were disabled. Camera editing filters were not applied. Images of the severed ear sourced from each mouse were captured immediately after euthanasia. Quantification of skin pigmentation was performed using ImageJ image processing software.

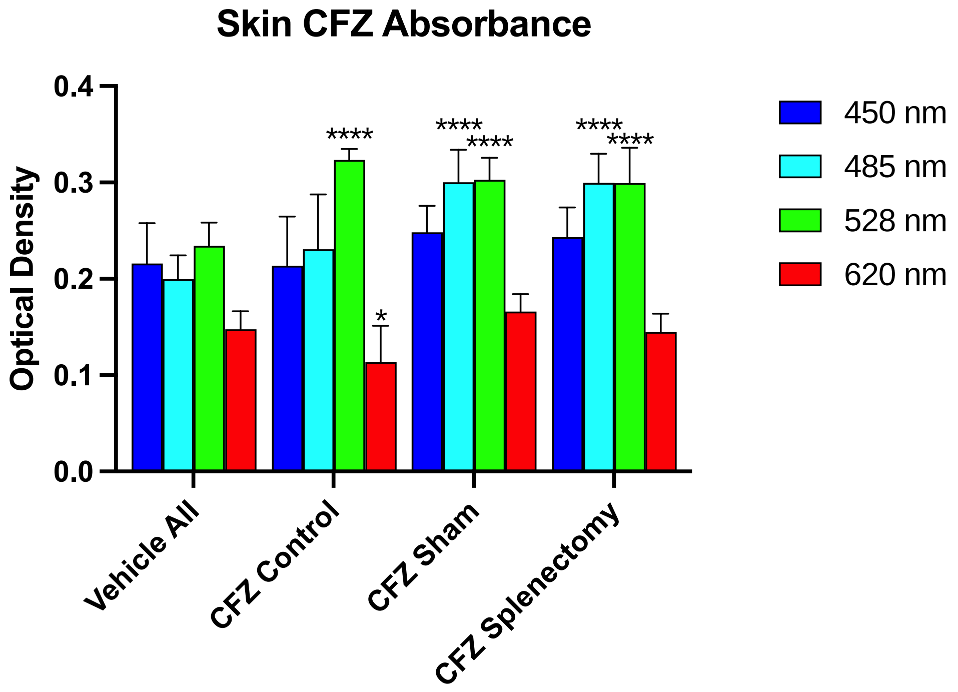

Figure S2: Quantitative analysis of CFZ concentration in the skin after 8 weeks of CFZ treatment. Vehicle All corresponds to the combined average of all vehicle-treated groups (vehicle control, vehicle sham, and vehicle splenectomy) Mean ± SD for each group is shown with four different band-pass filters. For a given wavelength, significant differences compared to the vehicle mice are marked. (blue = 450 nm filter; cyan = 485 nm filter; green = 528 nm filter; red = 620 nm filter).

($p < 0.05$, **** $p < 0.0001$, ANOVA single factor, Tukey's HSD).*

Significance was determined by performing ANOVA single factor with Tukey's HSD test in GraphPad Prism. The comparisons were made between the clofazimine treated splenectomy mice, clofazimine treated sham mice, clofazimine treated unoperated mice, and all vehicle treated mice regardless of operation at each wavelength. Significant differences in optical density were observed between vehicle-treated mice and clofazimine-treated mice regardless of

surgical procedure at 528 nm, indicating the presence of CLDIs. However, no significant absorbance differences were observed between splenectomy, sham, nor unoperated mice at 528 nm. Clofazimine-treated mice did have significant differences in absorbance at 485 nm depending on whether they received surgery, but the surgical operation performed – sham or splenectomy – did not produce significant differences in absorbance. Vehicle treated mice had significantly lower absorbances at 485 nm than either CFZ-treated sham or splenectomy mice, but not unoperated CFZ-treated mice. At 620 nm, significant differences in absorbance were observed between vehicle mice and CFZ-treated unoperated mice, and CFZ-treated unoperated mice and CFZ-treated sham mice. Significant differences in optical density were not observed between splenectomy and sham surgery mice at any wavelength. No significant differences between any groups were observed at 450 nm.

Clofazimine treatment produced differences in skin absorbance, in agreement with previous studies describing its pigmentation qualities. Across all wavelengths, the loss of spleen does not produce significant changes in CFZ-induced skin pigmentation when compared to general surgical trauma.

S.4 Study 2 Mouse Organ Weights and Drug Concentrations

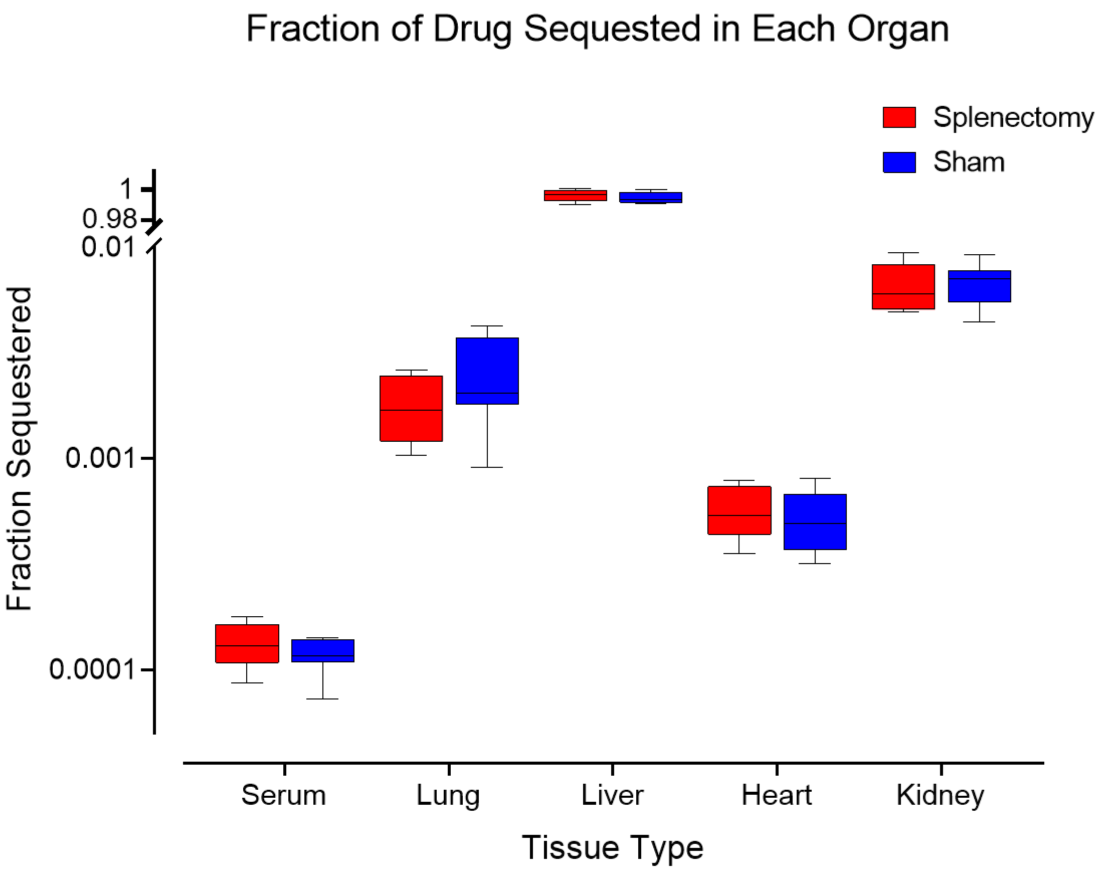

Figure S3: Drug sequestration in asplenic mice. The sham CFZ-treated mice (blue) compared to the asplenic CFZ-treated mice (red) show relative amounts of drug sequestered compared to the total amount of drug measured in these five tissue types. Values were calculated by dividing the mass in each tissue type by the sum of the mass sequestered in every organ (except the spleen in the sham group; Equation S1). 10th, 25th, 75th, and 90th percentiles are shown alongside the median values. No significant differences were found with a $p<0.05$. N=8 per group.

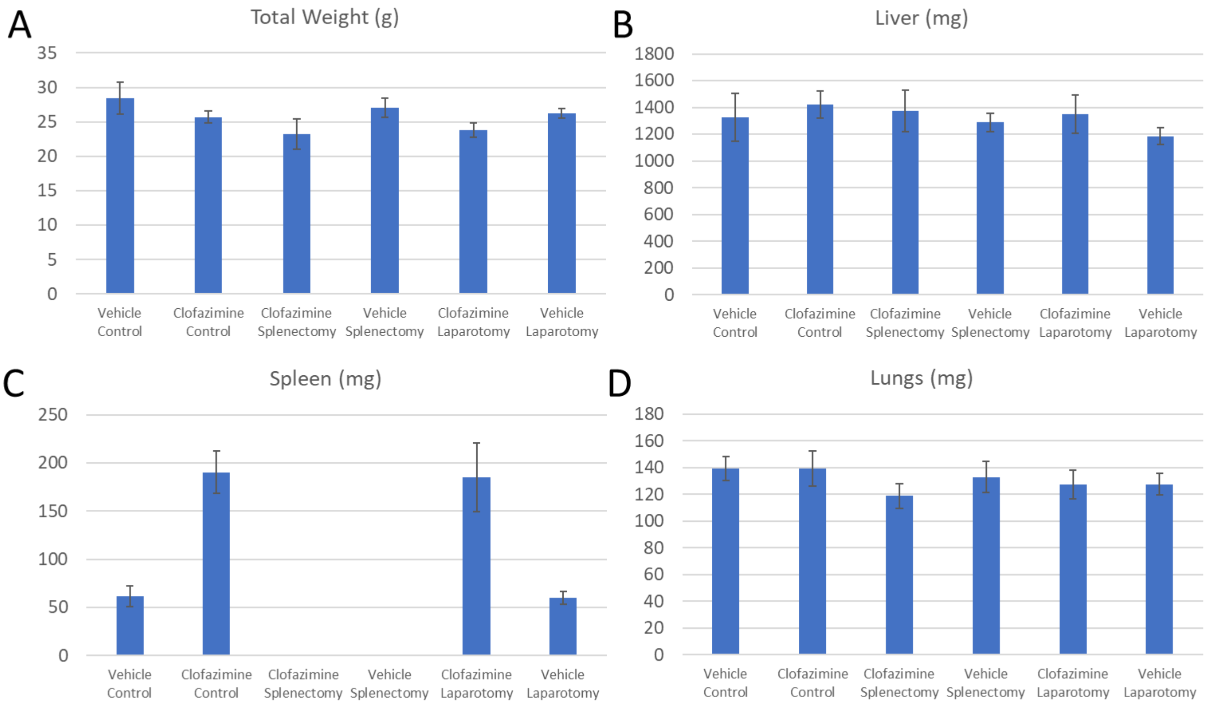

Figure S4: Weights of the primary drug sequestering organs in Study 2. No statistical significance ($p<0.05$) between CFZ laparotomy (sham surgery) and CFZ splenectomy (data are the mean [SD] of n=8/group)

When comparing total weight between the CFZ splenectomy group, and CFZ laparotomy group, no significance was found (Figure S4).

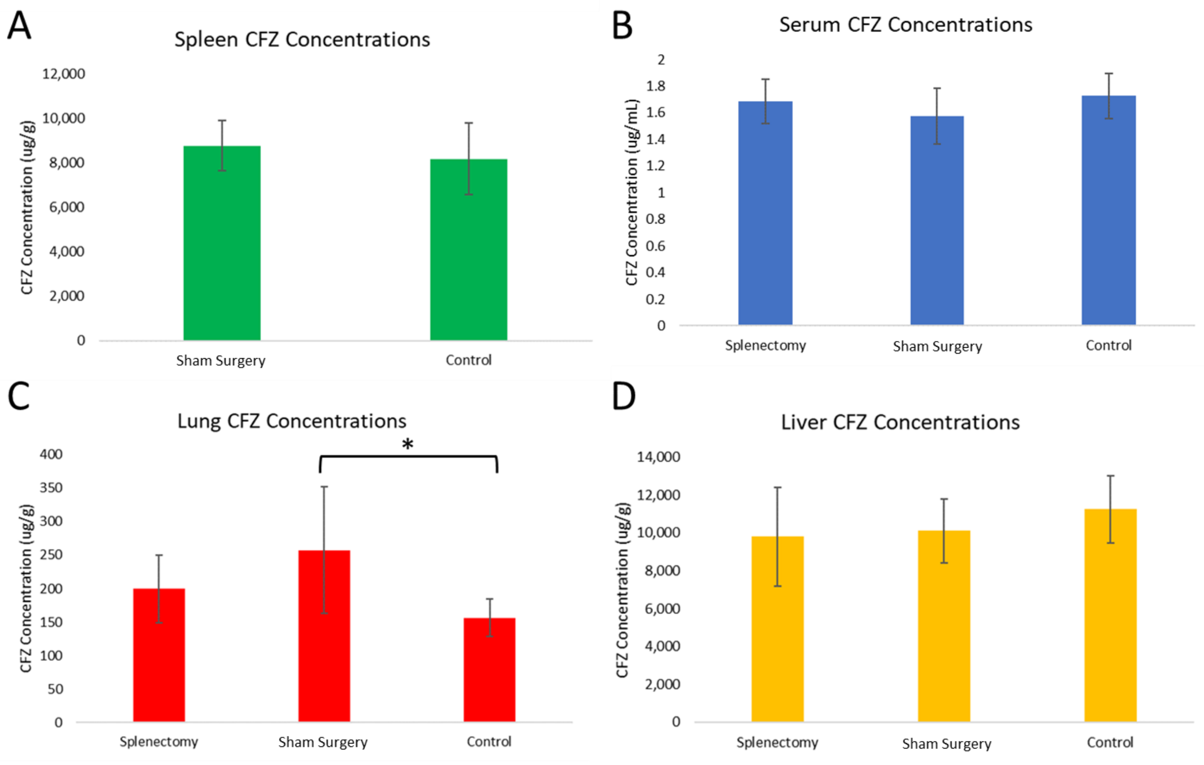

*Figure S5: CFZ concentration comparisons in the primary drug sequestering organs. * $p<0.05$ by ANOVA, n=8*

When comparing CFZ concentrations (Figure S5) and total CFZ sequestration (Figure S6) in the spleen, serum, lung, and liver, there were no differences in CFZ mass or CFZ concentrations between the splenectomy and sham surgery groups. However, there was a significant difference in lung concentration between the sham surgery group and control in the lung tissue.

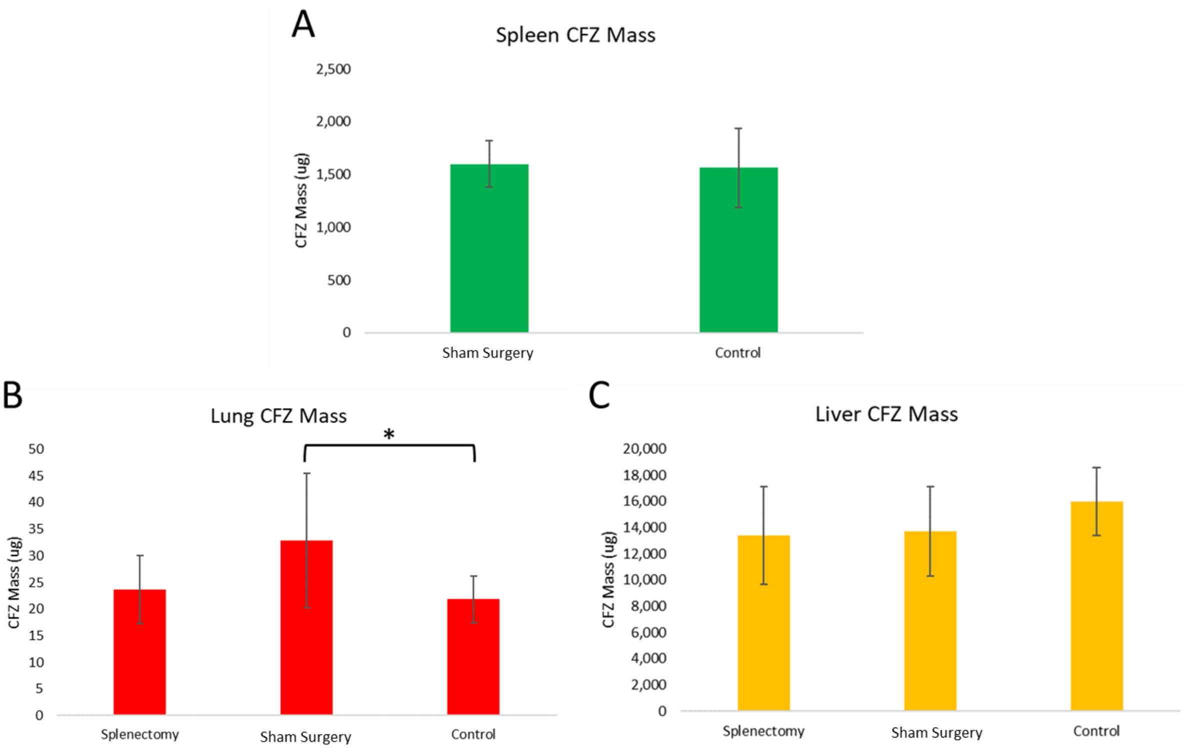

*Figure S6: Total CFZ mass comparisons among the primary drug sequestering organs. * p<0.05 by ANOVA, n=8*

S.5 Cytokine Density Values

Table S7. **Pixel densities of serum cytokines by experimental group.** The following table represents the average pixel density values for each cytokine in each of the six experimental groups of study 2, run in duplicate. All significant differences between CFZ Control vs. Vehicle Control, Vehicle Splenectomy vs. Vehicle Control, and Vehicle Sham vs. Vehicle Control as well as the relative effects of splenectomy on CFZ treatment are reported in the main manuscript.

Label	Vehicle Control		CFZ Control		CFZ Splenectomy		Vehicle Splenectomy		CFZ Sham		Vehicle Sham	
	Average	SD	Average	SD	Average	SD	Average	SD	Average	SD	Average	SD
Ref 1	1.00E+00	3.86E-02	9.32E-01	1.02E-01	9.47E-01	6.15E-02	9.25E-01	6.04E-02	9.76E-01	4.85E-02	9.46E-01	7.62E-02
BLC	5.65E-02	3.42E-02	7.61E-02	2.51E-02	1.15E-01	8.18E-02	4.97E-02	2.54E-02	6.84E-02	2.19E-02	7.15E-02	4.10E-02
C5	4.20E-01	9.83E-02	3.77E-01	9.16E-02	3.84E-01	5.16E-02	3.51E-01	6.20E-02	3.85E-01	8.84E-02	3.25E-01	4.69E-02
G-CSF	4.04E-03	5.65E-03	4.95E-03	2.35E-03	1.40E-02	1.71E-02	4.42E-03	8.50E-03	1.80E-03	1.59E-03	3.91E-03	2.70E-03
GM-CSF	2.46E-04	3.97E-03	-4.50E-04	1.20E-03	5.95E-04	2.32E-03	-1.25E-04	2.81E-03	1.08E-02	2.85E-02	2.11E-03	3.69E-03
ICAM-1	1.34E+00	2.24E-01	1.25E+00	2.04E-01	1.41E+00	2.78E-01	1.44E+00	2.12E-01	1.32E+00	2.06E-01	1.29E+00	9.16E-02
IFN-gamma	2.53E-02	1.57E-02	1.78E-02	4.18E-03	1.87E-02	3.55E-03	1.40E-02	1.22E-02	7.47E-03	4.72E-03	1.99E-02	4.88E-03
IL1-RA	4.03E-03	5.84E-03	1.80E-02	6.48E-03	1.48E-02	4.42E-03	4.13E-03	4.57E-03	6.91E-03	3.84E-03	5.50E-03	2.28E-03
IL-2	5.60E-03	7.49E-03	4.15E-03	3.50E-03	8.85E-04	1.96E-03	3.86E-03	4.50E-03	6.25E-04	9.85E-04	3.96E-03	1.79E-03
IL-1 alpha	1.12E-02	8.43E-03	9.14E-03	2.25E-03	1.05E-02	2.29E-03	8.27E-03	9.37E-03	3.20E-03	1.42E-03	1.00E-02	3.74E-03
IL-1 beta	4.08E-03	5.86E-03	3.84E-03	1.28E-03	2.79E-03	1.31E-03	3.95E-03	4.10E-03	1.09E-03	1.42E-03	4.21E-03	2.44E-03
I-309	3.62E-03	4.11E-03	6.09E-03	5.71E-03	3.08E-03	4.52E-03	1.77E-02	3.18E-02	1.23E-02	3.27E-02	3.88E-03	2.12E-03
Eotaxin	2.05E-03	2.23E-03	1.62E-03	1.18E-03	1.36E-03	2.42E-03	9.00E-04	3.00E-03	3.12E-04	1.06E-03	1.47E-03	1.36E-03
IL-3	3.71E-03	6.16E-03	2.15E-03	1.26E-03	2.85E-03	1.71E-03	9.78E-03	2.68E-02	1.11E-03	1.55E-03	3.65E-03	1.64E-03
IL-4	4.00E-03	5.77E-03	3.60E-03	3.21E-03	2.85E-03	1.24E-03	2.82E-03	3.08E-03	1.04E-02	2.55E-02	4.33E-03	2.69E-03
IL-5	1.88E-03	4.59E-03	6.09E-04	1.42E-03	-3.53E-04	2.14E-03	8.51E-04	5.36E-03	-7.52E-09	1.02E-03	5.91E-04	1.51E-03
IL-6	1.29E-03	3.12E-03	2.10E-03	2.59E-03	1.11E-03	2.72E-03	5.46E-03	9.54E-03	8.38E-04	1.29E-03	2.37E-03	3.54E-03

IL-13	1.02E-02	1.62E-02	7.89E-03	2.89E-03	5.18E-03	2.08E-03	3.54E-03	4.50E-03	1.02E-03	1.00E-03	6.73E-03	4.42E-03
IL-12 p70	2.91E-03	5.53E-03	1.57E-03	1.51E-03	6.79E-04	1.67E-03	1.36E-03	3.46E-03	3.43E-04	9.81E-04	1.81E-03	1.40E-03
IL-23	3.85E-03	4.44E-03	3.56E-03	1.78E-03	1.13E-03	2.07E-03	2.55E-03	3.50E-03	3.11E-04	7.44E-04	3.62E-03	2.36E-03
IL-27	1.88E-02	1.96E-02	1.24E-02	1.02E-02	1.66E-02	1.83E-02	9.50E-03	6.43E-03	3.93E-03	2.69E-03	9.91E-03	6.10E-03
IL-16	2.91E-02	1.20E-02	7.87E-02	3.38E-02	8.83E-02	1.97E-02	3.49E-02	2.73E-02	4.66E-02	2.44E-02	2.49E-02	6.99E-03
IL-17	7.35E-03	6.66E-03	5.89E-03	2.87E-03	3.99E-03	2.09E-03	6.37E-03	4.76E-03	1.25E-03	8.83E-04	6.25E-03	3.06E-03
IL-7	4.55E-03	4.91E-03	4.74E-03	2.30E-03	3.85E-03	1.90E-03	4.43E-03	4.25E-03	1.25E-02	3.31E-02	4.91E-03	2.07E-03
IL-10	7.67E-03	1.89E-02	1.03E-03	1.54E-03	-5.46E-04	1.63E-03	7.60E-04	3.29E-03	2.46E-04	7.33E-04	1.13E-03	1.80E-03
IP-10	9.03E-03	4.54E-03	9.50E-03	3.29E-03	9.18E-03	2.61E-03	5.09E-03	4.46E-03	4.02E-03	4.26E-03	1.01E-02	3.93E-03
I-TAC	3.52E-03	4.96E-03	1.11E-02	6.37E-03	8.42E-03	4.89E-03	5.75E-03	1.20E-02	7.70E-03	4.58E-03	4.45E-03	2.20E-03
KC	1.07E-02	5.86E-03	9.69E-03	2.56E-03	1.25E-02	3.99E-03	5.62E-03	3.57E-03	4.38E-03	3.51E-03	6.50E-03	2.85E-03
M-CSF	1.15E-01	3.94E-02	1.16E-01	5.01E-02	1.02E-01	1.04E-02	1.06E-01	3.62E-02	9.80E-02	4.21E-02	1.10E-01	9.29E-03
MIG	5.08E-03	5.89E-03	7.32E-03	3.37E-03	5.02E-03	3.31E-03	2.38E-03	4.55E-03	1.32E-03	1.18E-03	5.54E-03	2.93E-03
MIP-1 alpha	3.55E-03	5.19E-03	3.26E-03	1.94E-03	2.44E-03	1.74E-03	1.11E-03	3.12E-03	4.27E-04	7.15E-04	3.69E-03	2.74E-03
RANTES	1.43E-02	1.19E-02	1.07E-02	3.11E-03	7.28E-03	4.97E-03	7.01E-03	4.46E-03	2.06E-03	2.28E-03	1.19E-02	3.81E-03
SDF-1	4.81E-01	9.11E-02	4.20E-01	1.22E-01	4.29E-01	7.95E-02	5.49E-01	1.00E-01	4.38E-01	9.91E-02	4.77E-01	6.04E-02
MIP-1 beta	2.02E-03	4.39E-03	1.52E-03	1.55E-03	4.13E-04	1.64E-03	7.99E-04	1.96E-03	1.28E-02	3.57E-02	9.78E-04	2.50E-03
MIP-2	4.11E-03	5.00E-03	8.79E-03	2.06E-03	6.63E-03	2.35E-03	1.05E-03	3.40E-03	1.15E-03	1.15E-03	3.94E-03	1.59E-03
JE	1.56E-02	6.20E-03	2.00E-02	2.86E-03	2.04E-02	7.19E-03	8.87E-03	6.96E-03	8.80E-03	2.45E-03	1.31E-02	2.23E-03
MCP-5	2.87E-03	4.09E-03	1.74E-03	1.19E-03	8.03E-04	2.09E-03	5.60E-05	2.72E-03	2.17E-04	5.92E-04	2.42E-03	1.85E-03
TARC	2.44E-03	3.33E-03	1.16E-03	9.55E-04	3.15E-03	1.39E-03	-1.76E-04	2.66E-03	3.75E-04	8.39E-04	1.57E-03	1.48E-03
TIMP-1	7.23E-02	1.60E-02	4.61E-01	9.83E-02	5.58E-01	1.11E-01	6.55E-02	6.14E-02	4.77E-01	8.83E-02	5.49E-02	8.43E-03
TNF-alpha	8.42E-03	7.07E-03	1.54E-02	4.02E-03	1.00E-02	3.57E-03	5.73E-03	6.19E-03	4.94E-03	2.67E-03	8.26E-03	1.98E-03
TREM-1	4.48E-03	4.74E-03	1.11E-02	3.80E-03	9.19E-03	2.21E-03	1.56E-03	4.02E-03	2.24E-03	1.71E-03	5.33E-03	1.94E-03
Ref-2	1.03E+00	9.65E-02	1.04E+00	9.46E-02	1.05E+00	7.27E-02	1.07E+00	6.60E-02	1.04E+00	5.21E-02	1.11E+00	6.92E-02
Ref-3	9.69E-01	1.20E-01	1.03E+00	9.38E-02	1.01E+00	6.77E-02	1.00E+00	4.99E-02	9.83E-01	4.31E-02	9.47E-01	4.18E-02
Neg-Control	2.02E-19	3.63E-19	1.79E-19	4.83E-19	-1.02E-20	9.50E-19	7.79E-20	8.93E-19	1.36E-20	6.32E-20	-5.24E-19	6.08E-19

S.6 Supplemental Equations

Equation S1: Spleen-Independent Fraction of Drug Sequestered

$$FS(Organ) = \frac{CFZ\ Mass\ in\ Organ}{Total\ CFZ\ Mass\ -CFZ\ Spleen\ Mass}$$

S.7 References

1. Murashov, Mikhail D et al. "The Physicochemical Basis of Clofazimine-Induced Skin Pigmentation." *The Journal of investigative dermatology* vol. 138,3 (2018): 697-703. doi:10.1016/j.jid.2017.09.031
